# Phytofabrication and Characterisation of Zinc Oxide Nanoparticles Using Pure Curcumin

**DOI:** 10.3390/ph16020269

**Published:** 2023-02-10

**Authors:** Batoul Alallam, Abd Almonem Doolaanea, Mulham Alfatama, Vuanghao Lim

**Affiliations:** 1 Advanced Medical and Dental Institute, Universiti Sains Malaysia, Bertam, Kepala Batas 13200, Penang, Malaysia; 2 Department of Pharmaceutical Technology, Faculty of Pharmacy, Kolej Universiti Antarabangsa Maiwp, Taman Batu Muda, Batu Caves, Kuala Lumpur 68100, Selangor, Malaysia; 3 Faculty of Pharmacy, Universiti Sultan Zainal Abidin, Besut Campus, Besut 22200, Terengganu, Malaysia

**Keywords:** zinc oxide nanoparticles, antimicrobial, curcumin, green synthesis, drug discovery from natural products

## Abstract

Zinc oxide and curcumin, on their own and in combination, have the potential as alternatives to conventional anticancer drugs. In this work, zinc oxide nanoparticles (ZnO NPs) were prepared by an eco-friendly method using pure curcumin, and their physicochemical properties were characterised. ATR-FTIR spectra confirmed the role of curcumin in synthesising zinc oxide curcumin nanoparticles (Green-ZnO-NPs). These nanoparticles exhibited a hexagonal wurtzite structure with a size and zeta potential of 27.61 ± 5.18 nm and −16.90 ± 0.26 mV, respectively. Green-ZnO-NPs showed good activity towards studied bacterial strains, including *Escherichia coli*, *Staphylococcus aureus* and methicillin-resistant *Staphylococcus aureus*. The minimum inhibitory concentration of Green-ZnO-NPs was consistently larger than that of chemically synthesised ZnO NPs (Std-ZnO-NPs) or mere curcumin, advocating an additive effect between the zinc oxide and curcumin. Green-ZnO-NPs demonstrated an efficient inhibitory effect towards MCF-7 cells with IC_50_ (20.53 ± 5.12 μg/mL) that was significantly lower compared to that of Std-ZnO-NPs (27.08 ± 0.91 μg/mL) after 48 h of treatment. When Green-ZnO-NPs were tested against *Artemia* larvae, a minimised cytotoxic effect was observed, with LC_50_ being almost three times lower compared to that of Std-ZnO-NPs (11.96 ± 1.89 μg/mL and 34.60 ± 9.45 μg/mL, respectively). This demonstrates that Green-ZnO-NPs can be a potent, additively enhanced combination delivery/therapeutic agent with the potential for anticancer therapy.

## 1. Introduction

The rapid emergence and growth of nanobiotechnology has created a varied range of new applications for the biomedical and pharmaceutical industries [1,2,3,4]. Currently, metal oxide nanoparticles are becoming more popular owing to their useful physicochemical and biological characteristics [5]. Specifically, zinc oxide nanoparticles (ZnO NPs) are commonly used due to their broad range of applications in many fields. ZnO NPs possess potential biological applications, including antioxidant, antimicrobial and anticancer [6,7]. Their preparation process is simple, and they are considered biocompatible and safe materials [8]. Therefore, they are ideal for biomedical applications such as nanomedicine carriers, nanodiagnostics, biomolecular detection and luminescence materials [9]. Several physical and chemical synthesis methods have been used to synthesise ZnO NPs, including sol–gel [10], microwave-assisted [11], and thermal decomposition [12] methods; however, these techniques could produce toxic materials. The green production approach of ZnO NPs, which predominantly uses phytocompounds, is currently gaining popularity since it is a safer, low-cost, and environmentally friendly process.

In recent decades, functional foods and nutraceuticals have been widely investigated for the prevention and treatment of various diseases. Natural products play an essential role in the pharmaceutical sector as medications and supplements due to their multimechanistic biological activity, safety, and long-term availability. Plants are the most abundant source of bioactive natural compounds as well as a variety of macro- and micronutrients [13,14].

*Curcuma longa* (turmeric), a member of the ginger family (*Zingiberaceae*), is a short-stemmed perennial plant that grows naturally all over the Indian subcontinent and South East Asia [15]. It has been used for thousands of years as a remedy in traditional Indian and folk medicine for the cure of a large variety of illnesses, such as inflammation, infectious diseases, and gastric, hepatic, and blood disorders [16]. Curcumin (diferuloylmethane) is the main polyphenol isolated from the rhizomes of *Curcuma longa* [13]. It has a wide range of pharmacological effects, such as antioxidant, antibacterial, anticarcinogenic, and antiproliferative activities [17,18]. Curcumin can regulate the expression and the activity of various biological processes, which may explain its potential use in cancer chemotherapy [14,19]. It has the ability for the green synthesis of metal nanoparticles due to the presence of polyphenol acting as a reducing agent [20]. However, the application of curcumin has been limited in the production of ZnO NPs due to low water solubility and sensitivity to heat, light, and alkaline. Various methods have been reported for the green synthesis of ZnO NPs from curcumin using hydroxides or chemical mediators [21,22,23,24,25]. However, several disadvantages including time consumption [25], the toxicity of the chemicals used as a mediator [23,25,26], and the large size of ZnO NPs [21] are the major limitations of these methods.

Therefore, this study reported the synthesis of zinc oxide nanoparticles (Green-ZnO-NPs) which can be considered green, eco-friendly and safe for producing nanoparticles as an anticancer agent. Zinc acetate was used as the zinc precursor, while pure curcumin was used as the reducing agent. The prepared nanoparticles were characterised for their physicochemical properties, as well as their antioxidant activity. The antibacterial activity of Green-ZnO-NPs, as well as their anticancer activity against MCF-7 cells, were evaluated and compared to chemically synthesised zinc oxide nanoparticles (Std-ZnO-NPs) and mere curcumin. The impact of Green-ZnO-NPs on the ecotoxicity against brine shrimp larvae was also examined.

## 2. Results and Discussion

### 2.1. Synthesis of Green-ZnO-NPs

**Figure 1:**
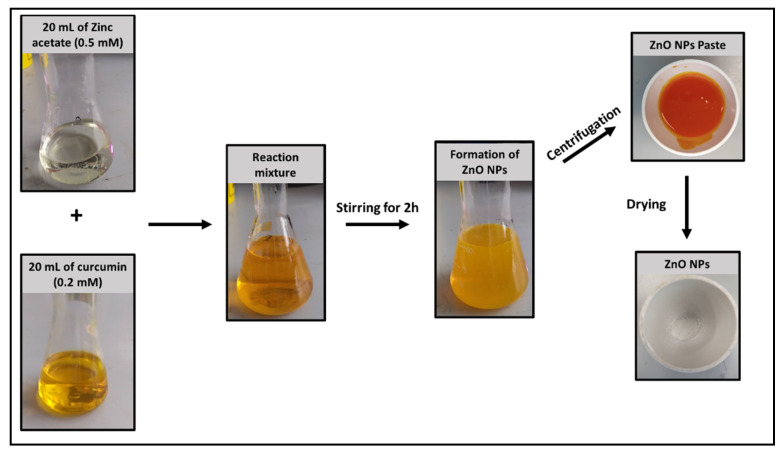
Synthesis scheme of Green-ZnO-NPs.

Green-ZnO-NPs were prepared by a green method using curcumin as a reductant and stabiliser. The change in the reaction mixture’s colour and the resulting orangish-white precipitate were used as indicators of the formation of the nanoparticles [27], as illustrated in Figure 1. Curcumin (diferuloylmethane) is a polyphenolic compound derived from the spices turmeric, consisting of bis-α, β-unsaturated β-diketone which exists in equilibrium with its enol tautomer [28]. Results of the NMR studies have confirmed that curcumin exists in solution in the form of the keto–enol tautomer [29]. The majority of the reductants have phenolic group or a β-diketone group in their structure [30]. Hence, the highest reducing ability of curcumin can be obtained when the phenolic hydroxyl group is sterically hindered by the introduction of two methyl groups at the ortho position of the benzene ring [31]. Therefore, the presence of two structural elements, namely, the β-diketone structure and the hydroxyl group at the ortho position in the aromatic ring, are the governing factors for the reducing potential of curcumin [31].

The possible mechanism for the formation of ZnO NPs is by the reaction of zinc acetate (precursor) with the polyphenols (from curcumin) to form an intermediate compound (zinc hydroxide). This compound is then transformed into zinc oxide mediated by the heat produced in the drying step, according to the following reactions:

Zn(CH_3_CO_2_)_2_ (aq) + 2R–OH (aq) → Zn(OH)_2_ (s) + 2R–CH_3_CO_2_ (aq)

Zn(OH)_2_ (s) + heat → ZnO (s) + H_2_O (v)

### 2.2. Characterisation of Green-ZnO-NPs

#### 2.2.1. Ultraviolet–Visible (UV–Vis)

**Figure 2:**
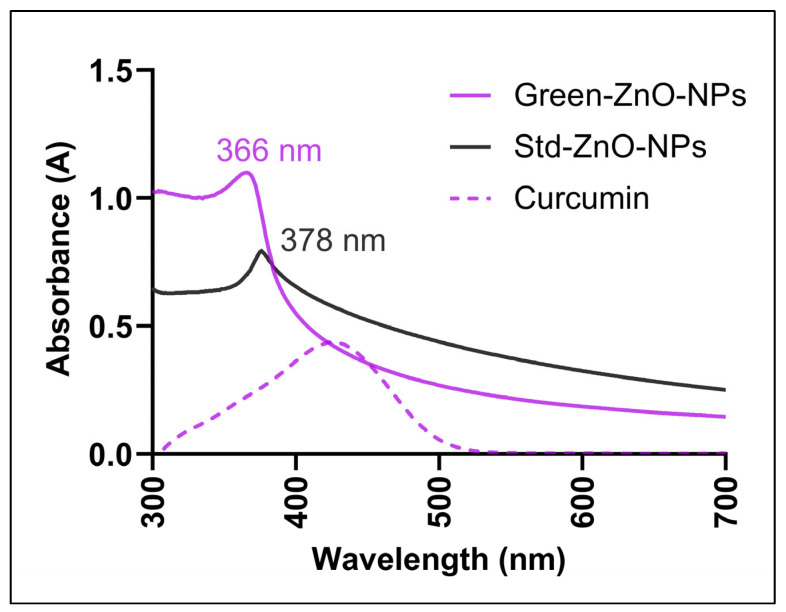
UV-Vis absorption spectra of the aqueous suspension of Green-ZnO-NPs (100 µg/mL), the aqueous suspension of Std-ZnO-NPs (100 µg/mL), and the ethanolic solution of curcumin (10 µg/mL).

The optical properties of Green-ZnO-NPs, Std-ZnO-NPs and curcumin were recorded from 300 to 700 nm using a UV–Vis spectrophotometer (Figure 2). Green-ZnO-NPs and Std-ZnO-NPs displayed an absorption peak at 366 nm and 376 nm, respectively. The peak at 366 nm has confirmed the successful synthesis of the nanoparticles with the aid of curcumin. It is claimed that ZnO NPs had a characteristic absorption peak at a range of 330–460 nm [32]. This peak is related to the intrinsic band-gap absorption of zinc oxide driven by UV-induced electron transitions (O2p → Zn3d) [33]. Curcumin exhibited a wide band with a maximum absorbance peak at 425 nm, which could be correlated to the low energy π–π* excitation of the curcumin [34]. The synthesis of ZnO NPs using curcumin may be elucidated by the capability to bioaccumulate metal ions, besides the ability to stabilise the process [35].

#### 2.2.2. Attenuated Total Reflectance-Fourier-Transform Infrared (ATR-FTIR) Analysis

**Figure 3:**
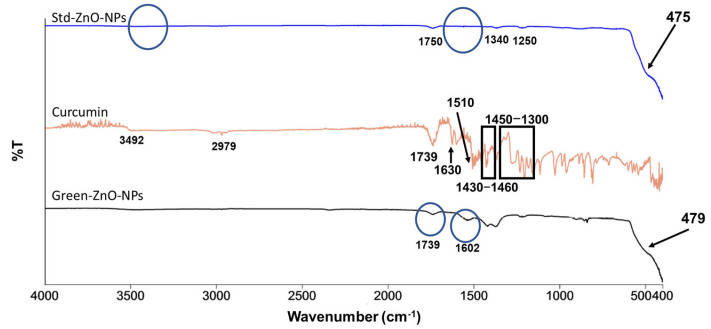
FTIR spectra of Std-ZnO-NPs (**up**), curcumin (**middle**), and Green-ZnO-NPs (**down**). The circles in Std-ZnO-NPs show that there were no peaks at 3500 cm^−1^ and 1600 cm^−1^, confirming the absence of hydroxyl groups on ZnO NPs surfaces.

FTIR analysis was conducted to examine the role of curcumin molecules in reducing and stabilising Green-ZnO-NPs. Std-ZnO-NPs showed the characteristic Zn–O bond at 475 cm^−1^ (Figure 3) [36], while no peaks were observed at 1600 cm^−1^, which represents the surface bending vibration of H-OH. The peak that appeared at 3500 cm^−1^ represents the stretching vibration of O-H, confirming the absence of hydroxyl groups on ZnO NPs surfaces, indicating that ZnO was formed rather than Zn(OH)_2_. Other peaks with a low intensity at 1250, 1340, and 1750 cm^−1^ could be correlated to the adsorbed carbonate moieties [37,38].

The band at 475 cm^−1^ in the Std-ZnO-NPs spectrum was shifted to 479 cm^−1^ and was less pronounced in the spectrum of Green-ZnO-NPs, proving the alternation of the Zn–O bond as it could interact with curcumin, and it is attributed to the vibration of hexagonal ZnO. The frequency region of curcumin phenolic vibrations was reported at 3595 cm^−1^; however, it was shifted to 3492 cm^−1^, which could be due to the intra- and intermolecular H-bonding in curcumin [39]. This band was not present in the spectrum of the Green-ZnO-NPs, demonstrating the absence of interaction between phenolic hydroxyls and ZnO.

The β-diketone group is one of the most prominent functional groups in curcumin molecules, with a high affinity for chelating metal ions [40,41]. Curcumin spectra showed a peak in the carbonyl region (1800–1650 cm^−1^), revealing that curcumin mainly exists in keto form. In the spectrum of Green-ZnO-NPs, the peak at 1739 cm^−1^ is associated with the asymmetric vibration of carbonyl in the keto form, while the symmetric mode was not observed. On the other hand, the band of enolic vibration (OH) appeared weak at 2979 cm^−1^, demonstrating the presence of both keto/enol forms in curcumin molecules. Accordingly, the β-diketone moiety most probably interacts (weakly or strongly) with zinc atoms at the bulk ZnO surface. The broadness and intensity of the enol peak are dependent on intramolecular hydrogen bond strength. As hydrogen bond strength rises, the intensity of the enol band decreases and its broadness increases [42]. The hydroxy and methoxy groups on the curcumin phenyl rings (electron-donor) are anticipated to cause a stronger hydrogen bonding. As a result, any reduction of the electronegativity of these groups via conjugating or bonding with other moieties could decrease the hydrogen bonding strength; consequently, this would allow the enol band to appear more clearly. Thus, the absence of a clearly defined enolic vibration band (OH) suggests that there was a weak complexation between zinc and curcumin at the methoxy group of phenolic rings. In the spectrum of curcumin, the peak at 1630 cm^−1^ has mixed C=O and C=C vibrations. In Green-ZnO-NPs, this band shifted to 1602 cm^−1^, which indicates strong coordination of the carbonyl moiety [43]. In the spectrum of curcumin, the band at 1510 cm^−1^ represents highly mixed CC=O, C=O and CC=C vibrations [39], while the peaks in the region of 1430–1460 cm^−1^ are attributed to the methyl vibration. Most peaks in the range of 1450–1300 cm^−1^ are mixed. Curcumin chelates with zinc via the carbonyl moity and a weak interaction between the curcumin phenyl ring (methoxy groups) and zinc moieties occurs.

#### 2.2.3. X-ray Powder Diffraction (XRD)

**Figure 4:**
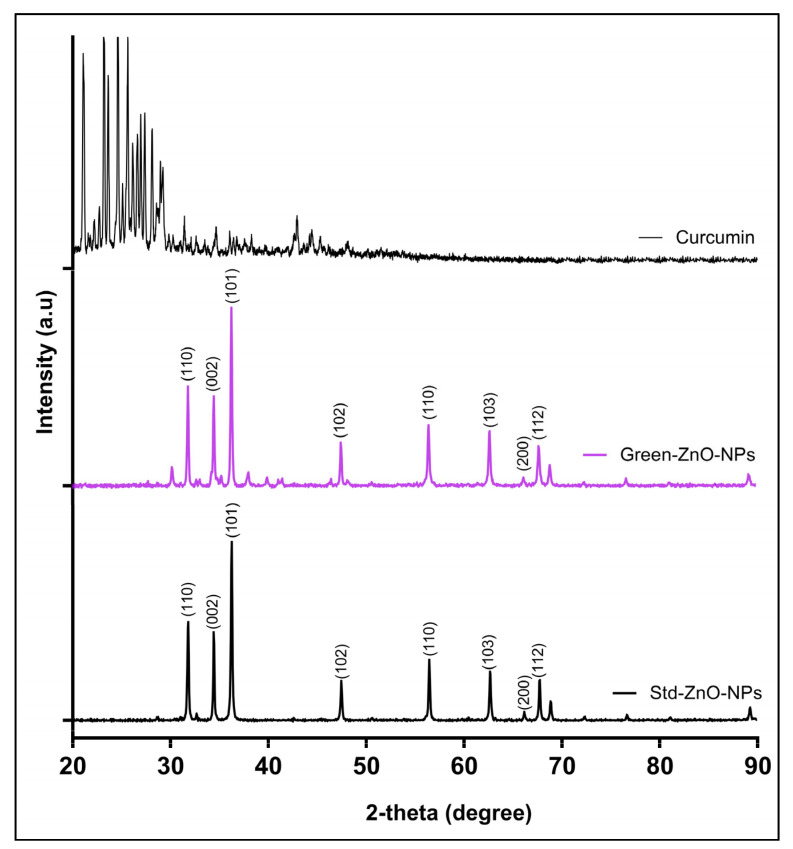
X-ray powder diffraction patterns of curcumin (**up**), Green-ZnO-NPs (**middle**) and Std-ZnO-NPs (**down**).

The crystallinity of Green-ZnO-NPs, Std-ZnO-NPs and curcumin was investigated by XRD. The experimental pattern of Green-ZnO-NPs (Figure 4) was consistent with the typical hexagonal zincite ZnO structure diffraction. All ZnO diffraction peaks were found in Green-ZnO-NPs at 31.85°, 34,50°, 36.20°, 47.50°, 56.45°, 62.71°, 67.73°, and 68.87°, corresponding to 110, 002, 101, 102, 110, 103, 200 and 112 lattice planes, respectively. These reported peaks comply with those obtained from the hexagonal phase of Std-ZnO-NPs with a wurtzite structure. All of the diffraction peaks are well correlated with the hexagonal phase of ZnO described in JCPDS card No. 36-1451 (space group *P63mc*). The diffraction peaks were sharp, highly intense and narrow, indicating the high crystallinity of Green-ZnO-NPs. No typical peaks of impurities or other phases of the zinc oxide were found, indicating the purity of the compound. The XRD pattern of curcumin exhibits peaks at the 2-theta range 20–30°, but these were not observed in Green-ZnO-NPs except for a peak at 30°.

#### 2.2.4. Surface Morphology Analysis

**Figure 5:**
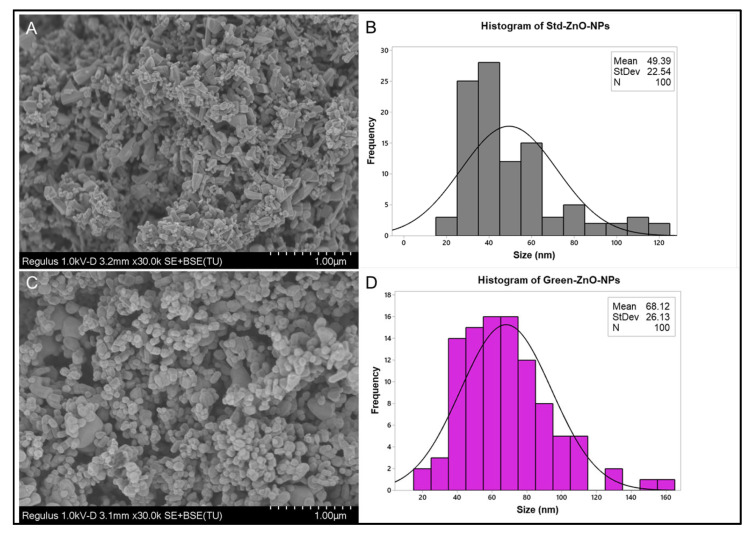
(**A**) Scanning electron microscopy images of Std-ZnO-NPs and (**B**) their corresponding histogram of particle size distribution (*n* = 100). (**C**) Scanning electron microscopy images of Green-ZnO-NPs and (**D**) their corresponding histogram of particle size distribution (*n* = 100).

**Figure 6:**
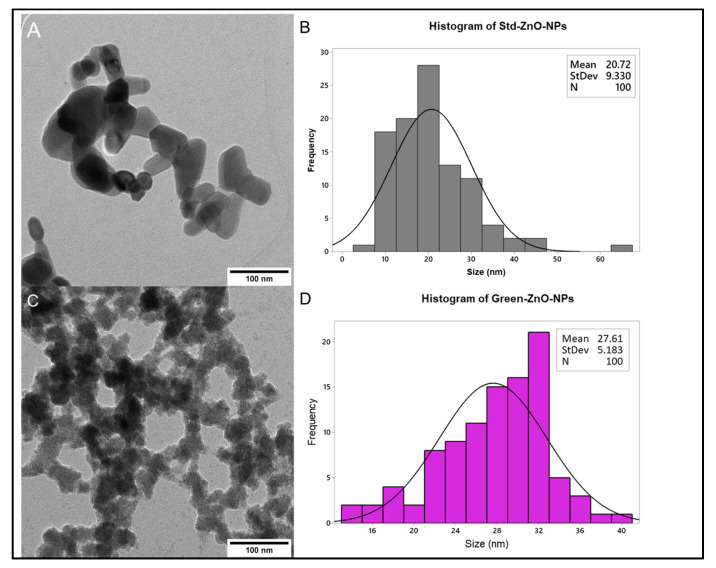
(**A**) Transmission electron microscopy images of Std-ZnO-NPs and (**B**) their corresponding histogram of particle size distribution (*n* = 100). (**C**) Transmission electron microscopy images of Green-ZnO-NPs and (**D**) their corresponding histogram of particle size distribution (*n* = 100).

Figure 5A,B show the typical hexagonal shape for Std-ZnO-NPs with an average size of 49.39 ± 22.54 nm. The morphology of Green-ZnO-NPs was predominantly grain-shaped or half-grain-shaped, while some were spherical (Figure 5C) with an average size of 68.12 ± 26.13 nm (Figure 5D). Using zinc acetate as a precursor, the ZnO nanoparticles developed slowly, forming small spherical structures that accumulate like bullets. The shape, size and size distribution of Green-ZnO-NPs and Std-ZnO-NPs were determined by using transmission electron microscopy (TEM) (Figure 6). Std-ZnO-NPs appeared mostly hexagonal in shape (Figure 6A), and the particle size was 20.72 ± 9.33 nm (Figure 6B). Green-ZnO-NPs mainly showed a grain shape with an average particle size of 27.61 ± 5.18 nm (Figure 6C,D). TEM indicates that both ZnO NPs showed aggregates.

The difference in particle sizes obtained by scanning electron microscopy (SEM) and TEM can be explained by the sample supplied for these analyses. For the SEM samples analysis, the powder of nanoparticles is placed onto an SEM stub, and it is later coated by platinum or conductors for nonconductive samples, while for the TEM samples analysis, the nanoparticles are dispersed in a solvent and then homogenised using a sonicator before the sample is placed onto a grid and the solution is allowed to air-dry to obtain a thinly sliced sample. Hence, TEM image analysis showed a smaller size of particles as the sample was homogenised, whereas the SEM images usually displayed agglomerated particles with larger sizes.

Several reports have been conducted to synthesise curcumin-conjugated zinc oxide nanoparticles with different methods and the particles were formed in various shapes and sizes. For instance, researchers have synthesised ZnO NPs complexed with curcumin to improve the potency and reduce the cytotoxicity, and the formed nanoparticles were spherical in shape [44]. Curcumin-doped ZnO nanospheres were effectively synthesised using zinc nitrate hexahydrate, with a size of 100–200 nm [45]. The size and shape of zinc oxide nanoparticles in these studies were dependent on many factors, such as the concentration and ratio of the reactants and pH.

#### 2.2.5. Particle Size and Zeta Potential

The dynamic light scattering method was utilised to observe the zeta potential, median hydrodynamic size and the size distribution of Green-ZnO-NPs and Std-ZnO-NPs. Green-ZnO-NPs had a hydrodynamic size of 171.67 ± 45.83 nm, compared to 909 ± 65.18 nm for the Std-ZnO-NPs (Table 1). It is possible that the large size is related to particle aggregation. The size variations can also be attributed to the availability of curcumin molecules on the surface of the nanoparticles. Curcumin may improve the colloidal stability of Green-ZnO-NPs; however, the particles were colloidally unstable, as evidenced by zeta values [46]. Further, the zeta potential value of Std-ZnO-NPs has been recorded at +2.76 ± 0.20 mV, as the wurtzite structure of ZnO NPs possessed a positive charge [47]. The zeta potential value for Green-ZnO-NPs was −16.90 ± 0.26 mV which is attributed to the presence of negatively charged hydroxyl groups of curcumin on the surface of zinc oxide nanoparticles (curcumin zeta potential −3.82 ± 0.31). The polydispersity index (PDI) of Std-ZnO-NPs was lower than the value of Green-ZnO-NPs, at 0.412 ± 0.039 and 0.698 ± 0.271, respectively. The broad particle size distribution is mostly due to particle size aggregation, which is mainly related to the electrostatic attraction of zinc oxide nanoparticles [48]. It was reported that the biosynthesised zinc oxide nanoparticles from *Curcuma longa* rhizomes exhibited a spherical shape with a diameter of 25 nm [49]. Another study synthesised zinc oxide nanoparticles using casein as a capping agent and conjugated curcumin on their surfaces with a diameter of 12.8 nm and a zeta potential of −23.9 mV, and the particles remained stable in the solution [50].

### 2.3. Antioxidant Activity

**Figure 7:**
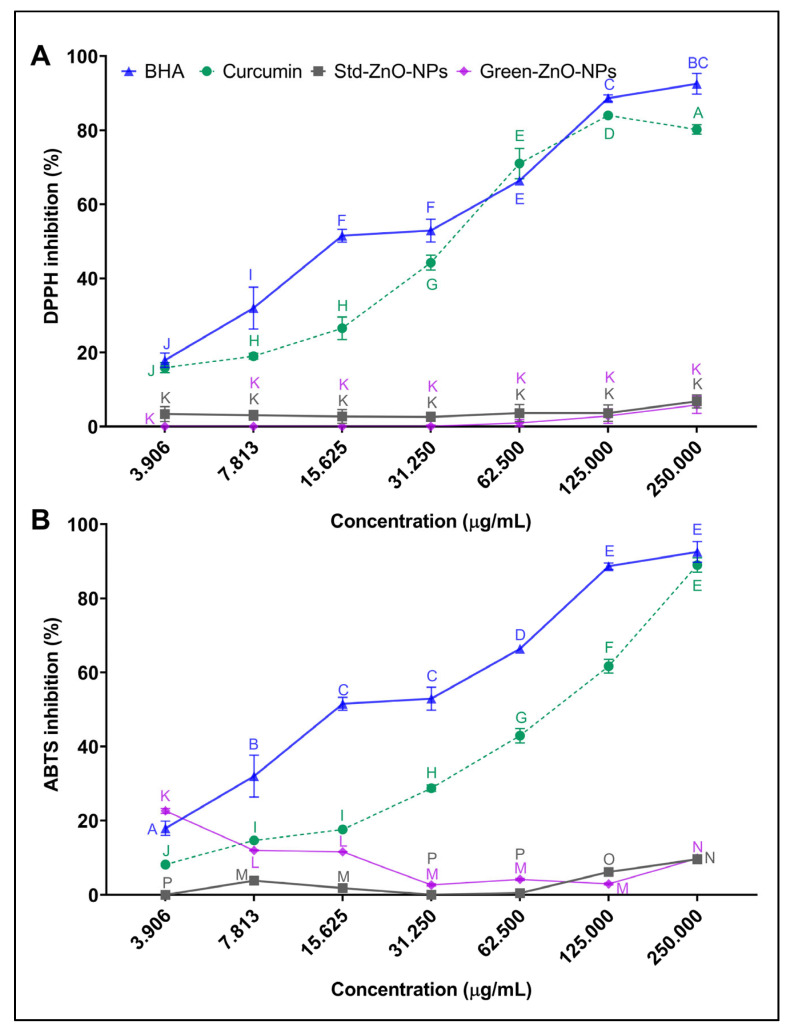
(**A**) DPPH and (**B**) ABTS antiradical activity of Green-ZnO-NPs, Std-ZnO-NPs and curcumin. Tukey post hoc multiple comparison tests show a significant difference (*p* < 0.05) between samples with different letters (mean ± SD; *n* = 3).

The antiradical activity of Green-ZnO-NPs, Std-ZnO-NPs, and curcumin was determined using 2,2-diphenyl-1-picrylhydrazy (DPPH) radical savaging activity (Figure 7A). Curcumin and butylated hydroxyanisole (BHA) exhibited a concentration-dependent scanning activity, which was augmented significantly with an increase in their concentrations. Nevertheless, BHA generally exhibited a significantly higher activity than curcumin and the IC_50_ value of curcumin (33.97 ± 3.20 µg/mL) was significantly higher than the value of BHA (20.04 ± 2.01 µg/mL). Curcumin was significantly more effective as a radical scavenger compared to both zinc oxide nanoparticles at all concentrations. Both zinc oxide nanoparticles (Green-ZnO-NPs and Std-ZnO-NPs) showed no IC_50_ values at the studied concentration range (>250 µg/mL). The reaction of DPPH involves transferring a hydrogen atom to the odd electron of the radical, causing a change in the colour of the sample [51,52]. FTIR analysis demonstrated that Green-ZnO-NPs and Std-ZnO-NPs were not rich in hydrogen (Figure 3), explaining their low radical scavenging activity. Moreover, their low scavenging activity could probably be due to the lower specific surface area and large particle size. It has been reported that the scavenging activity of nanocomposite containing gold nanoparticles is insignificant due to their lower specific surface area [53]. According to Stan et al. [54], the scavenging activity of zinc oxide nanoparticles is size-dependent, where the smallest size displayed the highest antiradical activity [54]. Zinc oxide nanoparticles using *Curcuma longa* rhizomes exhibited good antiradical activity, almost 70% at 200 µg/mL [49]. The antioxidant activity of curcumin-ZnO NPs demonstrates moderate activity by scavenging 70% at 2 mg/mL [45].

The 2,2′-azino-bis(3-ethylbenzothiazoline-6-sulfonic acid (ABTS) test measures the antioxidant ability to scavenge ABTS and produce ABTS^•+^. Radical scavenger acts as a representative for hydrogen donation [55]. Both BHA and curcumin showed a concentration-dependent ABTS inhibition effect (Figure 7B). BHA has shown considerably more efficacy in scavenging ABTS than curcumin at all studied concentrations, whereas ABTS inhibition is considerably increased at higher concentrations. Increasing the concentration from 3.9 to 250 µg/mL increased the scavenging activity of BHA and curcumin by 5.38- and 12.45-fold, respectively. Curcumin displayed an IC_50_ of 66.93 ± 3.64 µg/mL, which was significantly higher compared to that of BHA (20.04 ± 1.70 µg/mL). Both zinc oxide nanoparticles showed an IC_50_ > 250 μg/mL, and hence their antioxidant activity was significantly lower than that of curcumin.

### 2.4. Antimicrobial Activity

**Figure 8:**
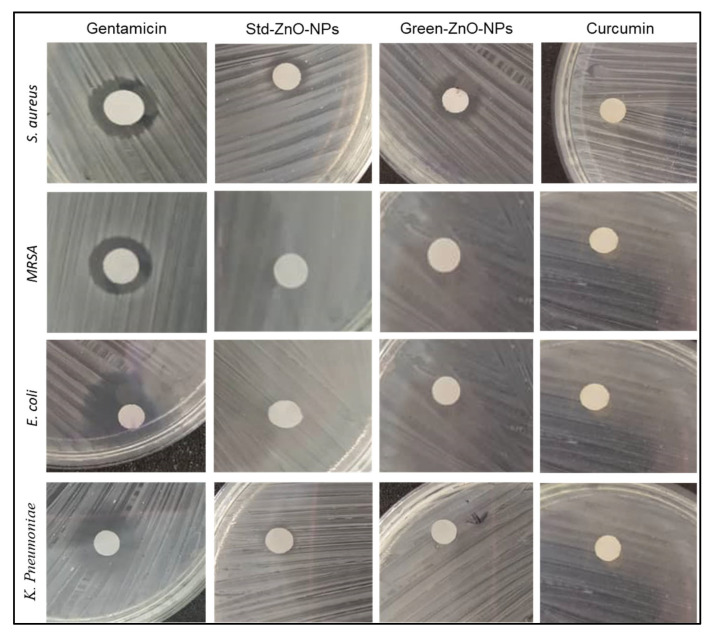
Zone of inhibitions of Gentamicin, Std-ZnO-NPs, Green-ZnO-NPs and curcumin against Gram-positive and Gram-negative bacteria.

The antibacterial properties of Green-ZnO-NPs, Std-ZnO-NPs, and curcumin against two Gram-negative [*Escherichia coli* (*E. coli*) and *Klebsiella pneumonia* (*K. pneumonia*)] and two Gram-positive [*Staphylococcus aureus* (*S. aureus*) and methicillin-resistant *S. aureus* (*MRSA*)] bacterial strains were measured as the diameter of zone of inhibition (ZOI), minimum inhibitory concentration (MIC), and minimum bactericidal concentration (MBC), using gentamicin as a positive control (Table 2). The ZOI varied based on the type of bacteria and the treatment (Figure 8). Gram-positive bacteria were more susceptible to Green-ZnO-NPs treatment than Gram-negative bacteria. The ZOI by Green-ZnO-NPs against *S. aureus* was 10.60 ± 0.10 mm, while no ZOI was observed against Gram-negative bacterial strains. Green-ZnO-NPs showed a greater ZOI compared to Std-ZnO-NPs against *S. aureus* (10.40 ± 0.15 mm), and their effect against *E. coli*, *K. pneumonia* and *MRSA* was significantly lower than that of gentamicin.

MIC is the lowest concentration that can reduce the growth of a microorganism after overnight incubation, whereas MBC is the lowest concentration that can prevent an organism from developing following a subculture of antibiotic-free media. MIC data showed that Green-ZnO-NPs exhibited bacteriostatic activity towards *E. coli*, *S. aureus* and *MRSA* at a concentration of 500 µg/mL; however, none of these concentrations demonstrated bactericidal activity (Table 2). Moreover, no MIC of Green-ZnO-NPs was observed against *K. pneumonia*. In addition, neither Std-ZnO-NPs nor curcumin showed antibacterial efficacy (MIC, MBC) against Gram-positive and Gram-negative bacteria.

The difference in the antibacterial activity between Green-ZnO-NPs and Std-ZnO-NPs could be due to the size of the nanoparticles. The activity of ZnO NPs is dependent on size and concentration. The concentration of ZnO NPs directly correlates with their antibacterial activity [56,57]. The higher concentration and smaller particle size are accountable for the higher antimicrobial activity of ZnO NPs [56,58]. Smaller ZnO NPs have a higher antibacterial efficacy owing to the large interfacial area that allows them to easily enter bacterial membranes. The MIC results revealed that the reduced size of the Green-ZnO-NPs displayed increased antibacterial propensity due to the large surface area to volume ratio, and subsequently, a high surface reactivity in comparison to Std-ZnO-NPs. Moreover, TEM analysis (Figure 6B,D) revealed more variability in the nanoparticle size distribution for Std-ZnO-NPs compared to Green-ZnO-NPs, which increases the probability of the Std-ZnO-NPs to congeal and form bigger particles, potentially reducing their antibacterial activity.

Moreover, this study found that *S. aureus* is more sensitive to ZnO NPs compared to *E. coli*, which is in accordance with a previous report by Reddy et al. [59]. Padmavathy and Vijayaraghavan [60] demonstrated that a higher degree of negatively charged free radicals caused cell damage and death in *S. aureus*. For Gram-negative bacteria strains, ZnO NPs must penetrate through the outer membrane alongside the peptidoglycan layer, while Gram-positive bacteria possess an outer membrane and a thick (30 µm) peptidoglycan layer [61]. Hence, Green-ZnO-NPs may function well as an antibacterial agent to combat both Gram-positive and Gram-negative bacteria.

The main mechanism of the antimicrobial activity of ZnO NPs remains controversial. The proposed mechanisms are described as follows: direct interaction between ZnO NPs and the bacteria cell walls, consequently the destruction of the integrity of bacterial cell membrane [56,62,63], followed by liberation of Zn^2+^ [64,65,66], and generation of ROS [67,68,69]. In this context, the antibacterial mechanism of ZnO NPs could be hypothesised to be the production of ROS such as hydroxyl radicals, hydrogen peroxide (H_2_O_2_), singlet oxygen, and zinc ions (Zn^2+^) released on the ZnO surface, which causes significant damage to the bacteria [57,70,71]. Bacterial growth is believed to be effectively inhibited by the production of H_2_O_2_ from the surface of ZnO [57]. According to certain reports, UV and visible light can activate ZnO, which results in the formation of electron–hole pairs (e^−^/h^+^). These holes break the H_2_O molecule into OH^−^ and H^+^ from the suspension of ZnO. Moreover, H^+^ react with the dissolved oxygen molecules to generate superoxide radical anions (O_2_^−^), which are then converted into hydrogen peroxide anions (HO_2_^−^) radicals. These hydroxyl radicals will collide with electrons to form HO_2_^−^, and will then react with H to form H_2_O_2_ molecules. Consequently, produced H_2_O_2_ molecules can enter the cell membrane and kill the bacterium [72,73]. Lattice defects play a significant part in limiting the e^−^/h^+^ pair recombination process, which reduces the likelihood of ROS generation [74]. Biogenic ZnO made from aqueous extracts includes several defects that could serve as trapping centres and prevent photoinduced e^−^/h^+^ pair recombination [75] resulting in biogenic ZnO NPs having greater antibacterial activity compared to that of chemical ZnO NPs.

Moreover, several reports have demonstrated that the antibacterial property of zinc oxide nanoparticles is highly influenced by particle morphology [76,77,78]. It was revealed that the activity of nanoparticles is reliant on their shapes in terms of their active facets. Different synthesis techniques are involved in various active facets of the nanoparticle. ZnO nanorods have both (111) and (100) facets, while ZnO nanospheres only contain (100) facets. It is stated that higher antibacterial activity is exhibited by high-atom-density facets along with (111) facets [79]. Moreover, it has been stated that the enhanced internalisation of ZnO NPs nanostructures by the polar facets has contributed to the development of antibacterial effects and that a greater proportion of polar surfaces have a greater number of oxygen vacancies. ZnO morphologies having highly exposed (0001) Zn-terminated polar facets could provide higher antibacterial action [80].

### 2.5. Anticancer Activity

**Figure 9:**
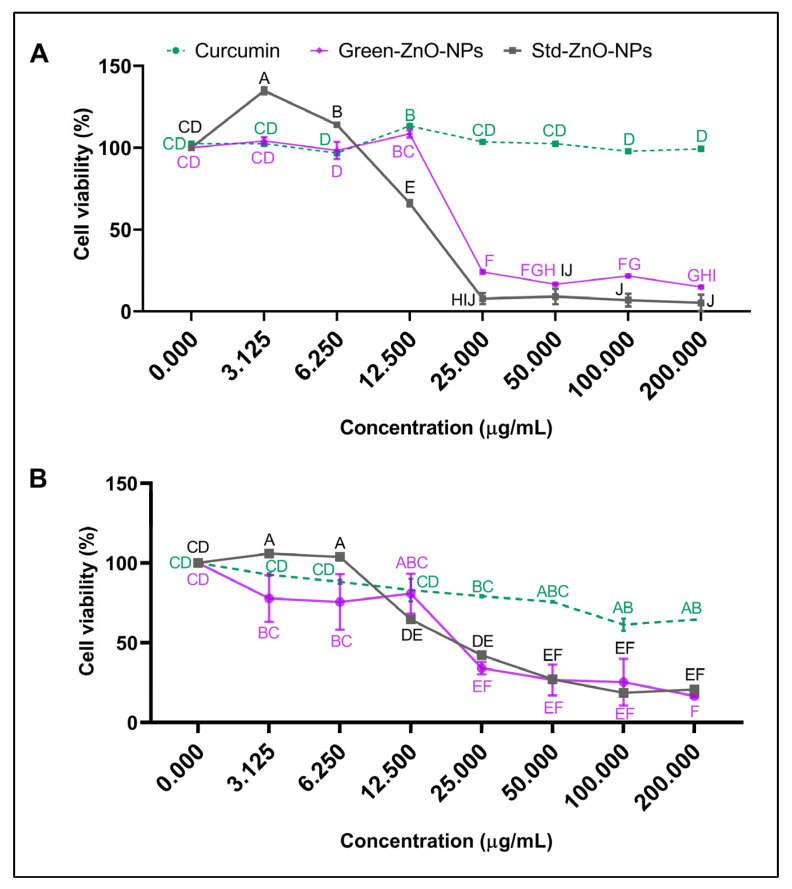
Anticancer activity of Std-ZnO-NPs, Green-ZnO-NPs and curcumin against MCF-7 human breast cancer cells after (**A**) 24 h and (**B**) 48 h of exposure. Tukey post hoc multiple comparison tests show a significant difference (*p* < 0.05) between samples with different letters (mean ± SD; *n* = 3).

The anticancer effect of Green-ZnO-NPs was assessed against MCF-7 breast cancer cells by MTT assay for 24 and 48 h and was compared to Std-ZnO-NPs and curcumin (Figure 9). The results demonstrated a significant decline in MCF-7 cell viability upon increasing Green-ZnO-NPs concentration (3.125–200 μg/mL) for both treatment periods. However, Std-ZnO-NPs exhibited potential inhibiting activity against MCF-7 cells, which was higher than that of Green-ZnO-NPs at a concentration ≥ 12.5 μg/mL for both treatment periods. Std-ZnO-NPs showed a lower IC_50_ than Green-ZnO-NPs after 24 h of treatment (14.08 ± 0.91 μg/mL and 23.54 ± 0.04 μg/mL, respectively). However, extending the treatment time to 48 h enhanced the anticancer activity of Green-ZnO-NPs, while reducing the activity of Std-ZnO-NPs and Green-ZnO-NPs displayed IC_50_ values of 27.08 ± 0.91 μg/mL and 20.53 ± 5.12 μg/mL, respectively. This could be attributed to cell duplication, as the cells might double when the dose is decreased, allowing them to recover from a toxic shock and continue to proliferate. As Green-ZnO-NPs were substantially smaller than Std-ZnO-NPs, an improved anticancer effect was observed, supporting the claim of the particle size effect. Moreover, Green-ZnO-NPs exhibited a synergistic anticancer effect of curcumin and ZnO NPs.

Curcumin exhibited no anticancer activity before 24 h of treatment as the viability of curcumin-treated cells was >95% at all concentrations tested (3.125–200 μg/mL), whereas mild anticancer activity was obvious after prolongation of the treatment time to 48 h. Various studies have demonstrated that curcumin induces apoptosis in cancerous cells by inhibiting several intracellular transcription factors and secondary messengers [81,82,83]. Nevertheless, the reason for the low cytotoxicity could be related to the low solubility of curcumin. A low cytotoxicity of free curcumin against MCF-7 cell lines was observed by Chen et al., which is probably due to the low bioavailability caused by poor water solubility [84].

In addition, zinc oxide nanostructures could be used to attack tumour cells, providing a potential target for the development of an antitumour agent [85]. The toxic response to biological systems of zinc oxide nanoparticles is due to their catalytic activity and band gap [86,87]. Previous reports suggest that cytotoxicity depends on the size, shape and capping agent used to synthesise zinc oxide nanoparticles [88]. Although the actual mechanism of the cytotoxicity of zinc oxide nanoparticles is still unknown, various theories have been proposed. The intracellular release of Zn^2+^, along with ROS generation, is the key mechanism driving zinc oxide nanoparticle cytotoxicity [8,89].

### 2.6. Artemia Larvae Lethality Bioassay

**Figure 10:**
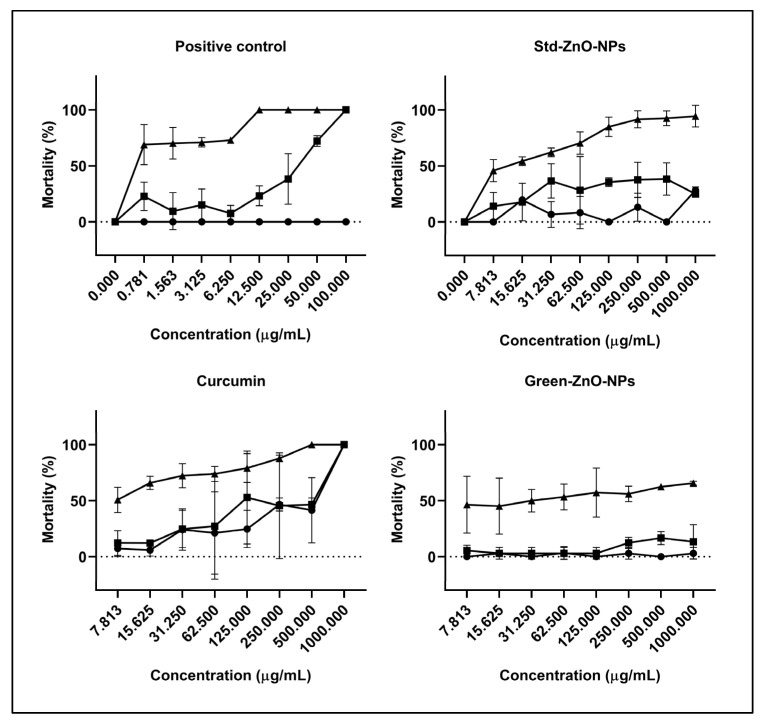
Mortality rates of *Artemia larvae* treated with potassium dichromate (positive control), Std-ZnO-NPs, Green-ZnO-NPs and curcumin at various concentrations after 4 (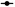), 8 (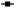), and 24 h (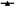).

Zero mortality was observed in the negative control, demonstrating that the deprivation of food had no lethal effect on *Artemia* larvae even after 24 h. The potassium dichromate showed a time-dependent effect, with no mortality observed after 4 h of exposure, while it showed a dose-dependent effect at 8 and 24 h (Figure 10). Although there was a substantial variation in mortality as the concentration increased (6.25 μg/mL–100 μg/mL), 100% mortality was reported using a concentration of 100 g/mL. However, approximately 30% of mortalities were observed at a concentration of 6.25 μg/mL after 8 h of exposure. The lethal effects were more apparent after extending the treatment time to 24 h. The mortality rate of larvae treated with 0.78 μg/mL was 69.05 ± 17.98%, while it increased to 100 ± 0.00% at 100 µg/mL, indicating that the LC_50_ concentration of potassium dichromate was 0.38 ± 0.27 μg/mL.

The mortalities of the larvae treated with Std-ZnO-NPs were raised by increasing the concentration of nanoparticles and increasing the incubation time (*p* < 0.05). The mortality rate between larvae treated with several concentrations of Std-ZnO-NPs did not change significantly after 4 and 8 h of exposure. The average mortality rate after 24 h of exposure to Std-ZnO-NPs ranged from 45.83 ± 9.79% (7.8 μg/mL) to 94.44 ± 9.62% (1000 μg/mL). The LC_50_ of Std-ZnO-NPs after 24 h of exposure was 11.96 ± 1.89 μg/mL. The mortality rate of larvae treated with Green-ZnO-NPs was lower than that of Std-ZnO-NPs. The lethal effects at 7.8 μg/mL were 0.00 ± 0.00%, 5.53 ± 4.79% and 46.44 ± 25.32% at 4, 8 and 24 h of exposure, respectively, and at 1000 μg/mL rose to 3.27 ± 5.66%, 13.33 ± 15.28% and 65.61 ± 1.72% at 4, 8, and 24 h of exposure, respectively. The LC_50_ of ZnO-Cur-NPs at 24 h of exposure was 34.60 ± 9.45 μg/mL. At doses larger than 62.5 μg/mL, Green-ZnO-NPs had less fatal effects than Std-ZnO-NPs of identical concentrations (*p* > 0.05), even though the effects were not significantly different at 24 h exposure time at concentrations ≤ 62.5 μg/mL (*p* < 0.05). Curcumin’s lethal effects followed the same temporal patterns as Std-ZnO-NPs, where the LC_50_ of curcumin at 24 h of exposure was 7.30 ± 2.57 μg/mL. The mortalities of the curcumin treatment group recorded within 4 and 8 h were higher than those of Std-ZnO-NPs and Green-ZnO-NPs at the same concentration.

Although zinc is a versatile trace element for physical organisms, excessive quantities have been linked to cellular harm. Various studies on protozoa [90] and microalgae [91] have been undertaken on the harmful effects of ZnO NPs, where the toxicity is related to the release of Zn^2+^ into the solution from the nanomaterials. On the contrary, studies on the effect of ZnO NPs on various organisms, such as *Danio rerio* embryos [92], *Daphnia magna* [93], and *Tigriopus japonicus* [94], showed that the presence of zinc ions could not account for their toxicity.

**Figure 11:**
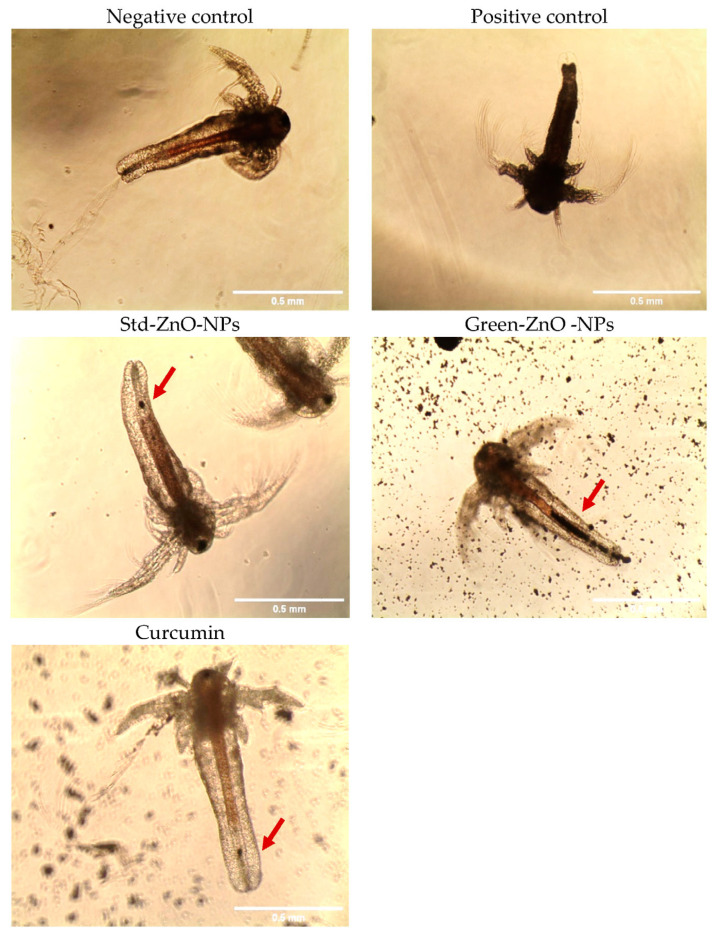
Morphology of treated (at their respective LC_50_) and untreated Artemia larvae. The red arrows indicate the swallowed treatment by *Artemia* larvae.

Furthermore, the *Artemia* larvae were imaged under the microscope after treatment with Green-ZnO-NPs, Std-ZnO-NPs and curcumin. Both Green-ZnO-NPs and Std-ZnO-NPs aggregated inside the gut of *Artemia* larvae, as clearly observed in Figure 11 (red arrow). *Artemia* larvae have a nonselective filter-feeding behaviour, and it consumes particles smaller than 50 µm. The aggregation level is influenced not only by the concentration of the nanoparticles but also by the quantity consumed by each individual larva at different concentrations. The control group’s guts were empty (the larvae did not exhibit any sign of aggregation, and the mouth and gut were transparent); however, the treatment group’s guts were completely loaded with nanoparticles, specifically Green-ZnO-NPs (Figure 11). The images also show that the ingested nanoparticles were removed by *Artemia* larvae. Moreover, no significant difference in the size of treated larvae compared to the untreated ones was observed (*p* > 0.005).

## 3. Materials and Methods

### 3.1. Materials

Zinc acetate dihydrate, foetal bovine serum (FBS) and Dulbecco’s modified eagle medium (DMEM) was purchased from Nacalai Tesque (Kyoto, Japan). Commercial zinc oxide nanoparticles (referred to as Std-ZnO-NPs in this study) with particle size < 100 nm, dimethyl sulfoxide (DMSO), 2,2-diphenyl-1-picrylhydrazyl (DPPH), 2,2′-azino-bis(3-ethylbenzothiazoline-6-sulfonic acid (ABTS) and 3-(4,5-dimethylthiazol-2-yl)-2,5-diphenyltetrazolium bromide (MTT) were received from Sigma Aldrich (Saint Louis, MO, USA). Curcumin (>97.0%) was received from Tokyo Chemical Industry (Tokyo, Japan). Bacterial culture media were purchased from Merck (Darmstadt, Germany). Gentamycin sulfate was received from Biobasic (Markham, ON, Canada). *Escherichia coli* [*E. coli* (ATCC 25922)], *Staphylococcus aureus* [*S*. *aureus* (ATCC 25923)], *Klebsiella pneumonia* [*k. pneumonia* (BAA-1705)] and methicillin-resistant *S. aureus* [*MRSA* (ATCC 33591)] were obtained from American type culture collection (ATCC).

### 3.2. Synthesis of Green-ZnO-NPs

Green-ZnO-NPs were synthesised following a method adopted from previous studies [95] with slight changes. Initially, a stock solution of curcumin was prepared using ethanol at 10 mM, and then it was diluted to 0.2 mM with ultrapure water. Zinc acetate was prepared as a stock solution (0.5 mM) using 20 mL of ultrapure water, and 20 mL of curcumin solution (0.2 mM) was added, followed by the mixing of these two solutions at room temperature (200 rpm, 2 h). Next, Green-ZnO-NPs were collected by centrifugation (3000× *g*, 3 min) and washed thrice with ultrapure water to eliminate the unreacted curcumin on the surface of zinc oxide, then dried at 70 °C for 24 h. Figure 1 illustrates the synthesis scheme of zinc oxide nanoparticles using pure curcumin.

### 3.3. Characterisation of Green-ZnO-NPs

#### 3.3.1. UV–Vis

A 100 µg/mL aqueous suspension of Green-ZnO-NPs and Std-ZnO-NPs was prepared and homogenised in a sonicator for 5 min. Curcumin solution was prepared using absolute ethanol (10 µg/mL). The suspensions of Green-ZnO-NPs and Std-ZnO-NPs, as well as curcumin solution, were examined by UV–Vis spectrophotometer (Shimadzu, Japan) over the range from 300 to 700 nm.

#### 3.3.2. ATR-FTIR Analysis

To evaluate the surface functionalisation, FTIR spectra of Green-ZnO-NPs, Std-ZnO-NPs, and curcumin were recorded using a Perkin Elmer FTIR spectrophotometer (Norwalk, CT, USA) at a wavelength of 4000 to 400 cm^−1^ [96].

#### 3.3.3. XRD

The crystalline structure of Green-ZnO-NPs, Std-ZnO-NPs, and curcumin was characterised by an X-ray diffractometer using 40 kV/40 mA current with Co-Kα radiation. The samples were scanned in the 20° to 90° 2-theta range.

#### 3.3.4. Surface Morphology Analysis

The primary size and morphology of Green-ZnO-NPs and Std-ZnO-NPs were evaluated using Libra 120 TEM (Zeiss, Oberkochen, Germany). Green-ZnO-NPs and Std-ZnO-NPs were dispersed at a concentration of 100 µg/mL in ultrapure water and homogenised in a sonicator for 5 min. A volume of 10 μL of Green-ZnO-NPs suspension was loaded onto a carbon-coated copper grid. The grid was dried for 10 min before it was examined using TEM. The histogram of particle size distribution was generated from three microscopy images by measuring the diameter of 100 nanoparticles using ImageJ software. Moreover, SEM (Hitachi-Regulus, Tokyo, Japan) was conducted to identify the surface morphology of Green-ZnO-NPs and it was compared to Std-ZnO-NPs. The SEM samples were prepared by placing the nanoparticles above the SEM carbon-coated stub, followed by removing the excessive nanoparticles with an air dust blower. The samples proceeded for imaging without coating.

#### 3.3.5. Particle Size and Zeta Potential

Green-ZnO-NPs and Std-ZnO-NPs had their hydrodynamic particle size, size distribution, and zeta potential recorded by a Zetasizer Nano ZS (Malvern Instruments Ltd., Malvern, UK). The analysis was performed for a duration of 10 s, 12–15 times, at 25 °C. An aqueous suspension of Green-ZnO-NPs and Std-ZnO-NPs was prepared at a concentration of 100 µg/mL and was homogenised in the sonicator for 5 min. Curcumin solution was prepared using absolute ethanol at a stock concentration of 10 mg/mL, then it was diluted with ultrapure water to 10 µg/mL, and its zeta potential was recorded.

### 3.4. Antioxidant Activity

DPPH radical scavenging activity of the aqueous suspension of Green-ZnO-NPs was tested and compared with Std-ZnO-NPs and curcumin, following a previous report with minor modifications [97]. In brief, 150 μL of 100 µM DPPH (in methanol) was mixed with 50 μL of each concentration (3.906, 7.813, 31.625, 62.5, 125, 250, and 500 µg/mL) of Std-ZnO-NPs, Green-ZnO-NPs and curcumin (dissolved primarily in absolute ethanol at 10 mg/mL concentration then diluted in ultrapure water), and then incubated for 30 min. A plate reader was used to record the optical density at 517 nm. The percentage of DPPH inhibition was calculated by subtracting the sample absorbance from the absorbance of the control by dividing the control absorbance multiplied by 100. BHA and ultrapure water were utilised as positive and negative controls, respectively.

Moreover, ABTS radical scavenging activities of Green-ZnO-NPs, Std-ZnO-NPs and curcumin were determined based on a previous report with minor changes [97]. Briefly, the ABTS working solution was made by mixing a volume of ABTS solution (7 mM) with a volume of potassium persulfate (2.45 mM). The prepared solution was stored for 16 h at room temperature. The ABTS working solution was diluted with ethanol until reaching an absorbance of 0.7 at 734 nm. A volume of 50 μL for each concentration (3.906, 7.813, 31.625, 62.5, 125, 250, and 500 µg/mL) of Green-ZnO-NPs, Std-ZnO-NPs and curcumin was mixed with 150 μL of ABTS working solution, followed by 6 min of incubation. Then, the optical density was recorded at 734 nm. The ABTS inhibition percentage was determined by subtracting sample absorbance from the absorbance of control by dividing the control absorbance multiplied by 100. BHA and ultrapure water were utilised as positive and negative controls, respectively.

### 3.5. Antibacterial Activity

The antibacterial properties of Green-ZnO-NPs, Std-ZnO-NPs and curcumin were tested against two Gram-negative bacterial strains (*E. coli*, *K. pneumonia*) and two Gram-positive bacterial strains (*S. aureus* and *MRSA*) using disc diffusion and broth microdilution assays.

#### 3.5.1. Preparation of Inoculum

A lawn culture prepared from each bacterial strain was prepared by subculturing a fresh 100 μL of bacterial solution (having 10^5^ CFU/mL, complying with McFarland 0.5) on MHA, which was incubated overnight at 37 °C.

#### 3.5.2. Disc Diffusion Method

The experiment was performed as described in a previous report by Chiu et al. [98]. The zones of inhibition of Green-ZnO-NPs were tested against the mentioned bacteria and compared with that of Std-ZnO-NPs and curcumin. Each bacterial strain was swabbed on the MHA plates. A volume of 30 µL of Green-ZnO-NPs and Std-ZnO-NPs suspensions (1 mg/mL), as well as curcumin solution, was applied to sterile discs with a diameter of 9 mm. Next, the discs were placed on swabbed plates. A clear zone of inhibition was measured after incubation and expressed in millimetres. Gentamicin solution (1 mg/mL) and ultrapure water were utilised as positive and negative controls, respectively.

#### 3.5.3. Broth Microdilution Assay

The experiment was performed as mentioned by Chiu et al. [98]. To assess the minimum inhibitory concentration (MIC), 180 μL of bacterial inoculum suspension was mixed with 20 μL of the Green-ZnO-NPs, Std-ZnO-NPs and curcumin at various concentrations (3.906, 7.813, 31.625, 62.5, 125, 250, 500, and 1000 µg/mL) in a 96-well plate, followed by 24 h of incubation at 37 °C. Gentamicin solution (1 mg/mL) and ultrapure water were utilised as positive and negative controls, respectively. Afterwards, 50 μL of MTT solution (500 μg/mL) was introduced to each well, followed by 30 min of incubation. The existence of a purple colour indicated the presence of viable bacteria. To determine the minimum bactericidal concentration (MBC), 10 µL of the wells that did not have a purple colour were streaked on MHA plates, followed by incubation at 37 °C for 24 h.

### 3.6. Anticancer Activity

MCF-7 cells were maintained in a complete culture medium (DMEM supplemented with 10% FBS) in a 5% CO_2_ incubator at 37 °C. The anticancer effect of Green-ZnO-NPs was assessed against MCF-7 cells according to a previous method [99] with minor changes and was compared to Std-ZnO-NPs and curcumin. Briefly, 1 × 10^4^ cells/well were seeded in a 96-well plate, grown overnight, and then treated with various concentrations (3.125, 6.25, 12.5, 25, 50, 100, and 200 µg/mL) of Green-ZnO-NPs, Std-ZnO-NPs and curcumin. After 24 or 48 h treatment exposure, the media were replenished with 10 μL of MTT (500 µg/mL) reagent and 90 μL of a fresh medium and kept in the incubator for a further 4 h. The medium was replaced with 100 µL of DMSO in each well of the plate, and the optical densities were measured at 570 nm. The percent of cell viability was calculated by dividing the absorbance from the treated cell over the absorbance from the untreated cell after blank subtraction and multiplying by 100. The IC_50_ after treatment were calculated for both time periods.

### 3.7. Artemia Larvae Lethality Bioassay

The hydration of brine shrimp (*Artemia franciscana*) eggs was first undertaken in ultrapure water overnight at 4 °C, followed by the collection and washing of the sinking cysts. Deionised water was used to make 3% *w/v* saltwater (without iodine), which was then filtered through 30 µm Millipore cellulose filters. Approximately 2 g of previously cleansed cysts were added to 1 L of salt water and a steady fluorescent bulb (1500 lux daylight) at 30 ± 1 °C [100]. Artemia larvae hatched under these conditions in less than 24 h.

The toxic effect of Green-ZnO-NPs, Std-ZnO-NPs, and curcumin on larvae mortality was investigated as described by Ates et al. [100]. The experiment was carried out on a 12-well plate and lasted 4, 8, and 24 h. A volume of 1 mL of saltwater with the addition of the required concentration of Green-ZnO-NPs, Std-ZnO-NPs, and curcumin (7.813, 31.625, 62.5, 125, 250, 500, and 1000 µg/mL) with around 15 larvae (<24 h old) were transferred to each well. The Artemia larvae were incubated with the treatment for 24 h, and then the mortality rate and half maximum lethal concentration (LC_50_) values were determined. Potassium dichromate at a concentration range of 0.781, 3.163, 6.250, 12.5, 25, 50, and 100 µg/mL was utilised as a positive control. The morphological variations in the treated and untreated brine shrimp larvae were observed using an inverted optical microscope at the treatment LC_50_ value (Kenis, 10×).

### 3.8. Statistical Analysis

All data in this research were collected from three independent biological replicates and presented as mean ± standard deviation (SD). Prism pad software was used to compute the IC_50_ and LC_50_ values. Significant differences between the means were analysed by one-way analysis of variance (ANOVA) in Minitab software, followed by Tukey post hoc multiple comparison tests. *P* less than 0.05 indicates significance.

## 4. Conclusions

In this research, the biosynthesis of ZnO NPs using curcumin was described, and their physicochemical properties were characterised. ATR-FTIR spectra have confirmed the role of curcumin in the formation of Green-ZnO-NPs. Moreover, Green-ZnO-NPs demonstrated a strong crystallinity property due to the hexagonal wurtzite structure. The produced NPs were grain-shaped in SEM and TEM microscopy images, with some of them having a spherical shape. They possessed a mean size of 27.62 ± 5.18 nm and a zeta potential of −16.90 ± 0.26 mV, respectively. Furthermore, the Green-ZnO-NPs showed a poor antioxidant effect using ABTS and DPPH tests. Even though Green-ZnO-NPs possessed weak antimicrobial activity, they exhibited high effectiveness in inhibiting MCF-7 breast cancer cells. MTT results suggest that Green-ZnO-NPs were more potent anticancer agents than chemically synthesised Std-ZnO-NPs and less toxic to *Artemia* larvae. Thus, it is envisaged that Green-ZnO-NPs could be a promising agent for anticancer applications.

## Figures and Tables

**Table 1:** Median hydrodynamic size, zeta potential and polydispersity index values of Green-ZnO-NPs, Std-ZnO-NPs and curcumin. Tukey post hoc multiple comparison tests show a significant difference (*p* < 0.05) between samples with different superscript letters (mean ± SD; *n* = 3).

	Zeta Potential (mV)	PDI	Particle Size (nm)		
			DLS	SEM	TEM
Std-ZnO-NPs	+2.76 ± 0.20 ^a^	0.412 ± 0.039 ^a^	909 ± 65.18 ^a^	48.98 ± 24.51 ^a^	49.39 ± 22.54 ^a^
Green-ZnO-NPs	−16.90 ± 0.26 ^b^	0.698 ± 0.271^a^	171.67 ± 45.83 ^b^	68.12 ± 26.13 ^a^	27.61 ± 5.18 ^a^
Curcumin	−3.82 ± 0.31 ^c^	-	-	-	-

**Table 2:** Zone of inhibitions, minimum inhibitory concentration, and minimum bactericidal concentration of Std-ZnO-NPs, Green-ZnO-NPs, and curcumin against Gram-positive and Gram-negative bacteria. Tukey post hoc multiple comparison tests show a significant difference (*p* < 0.05) between samples with different letters (mean ± SD; *n* = 3).

Microorganism	Gentamicin	Std-ZnO-NPs	Green-ZnO-NPs	Curcumin
	ZOI (mm)			
*E. coli*	22.87 ± 0.30 ^a^	-	-	-
*K. pneumonia*	11.63 ± 0.10 ^b^	-	-	-
*MRSA*	11.20 ± 0.06 ^c^	-	-	-
*S. aureus*	15.7 ± 0.04 ^d^	10.40 ± 0.15 ^e^	10.60 ± 0.10 ^e^	-
	MIC (µg/mL)			
*E. coli*	1.95	-	500	-
*K. pneumonia*	15.63	-	-	-
*MRSA*	7.81	-	500	-
*S. aureus*	0.98	-	500	-
	MBC (µg/mL)			
*E. coli*	7.81	-	-	-
*K. pneumonia*	31.25	-	-	-
*MRSA*	31.25	-	-	-
*S. aureus*	1.95	-	-	-
Indicates no activity				

## Data Availability

All data is comprised within this manuscript.
